# Supplementary material for: HaloTrace: A spatiotemporally precise fluorescent readout of blood-brain barrier permeability in mice
Source: bioRxiv. 2025 Oct 31:2025.10.30.685531. Preprint. [Version 1] doi: 10.1101/2025.10.30.685531 (PMC12636304; doi:10.1101/2025.10.30.685531)
Supplement: Supplement 1 [file NIHPP2025.10.30.685531v1-supplement-1.pdf]

bioRxiv preprint doi: https://doi.org/10.1101/2025.10.30.685531; this version posted October 31, 2025. The copyright holder for this preprint (which was not certified by peer review) is the author/funder, who has granted bioRxiv a license to display the preprint in perpetuity. It is made available under aCC-BY 4.0 International license.

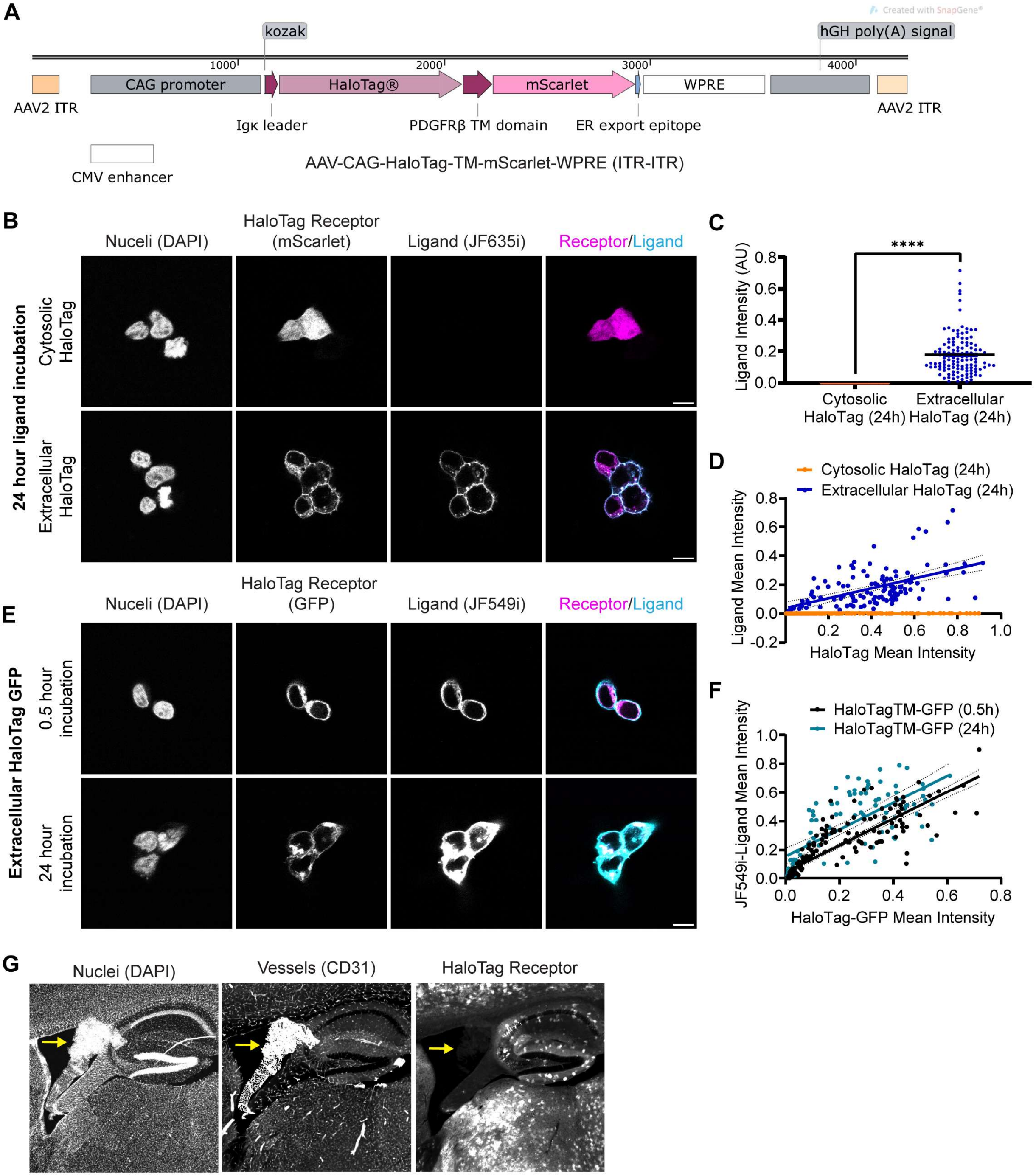

Supplementary Figure 1. Further characterization of HaloTrace components.

(a) Map of the AAV plasmid insert containing the CDS of extracellular membrane-targeted HaloTag fused to mScarlet. **(b)** 63x magnification confocal images of HEK cells transfected with cytosolic-targeted HaloTag-mScarlet or Extracellular HaloTagTM-mScarlet (magenta) incubated

bioRxiv preprint doi: https://doi.org/10.1101/2025.10.30.685531; this version posted October 31, 2025. The copyright holder for this preprint (which was not certified by peer review) is the author/funder, who has granted bioRxiv a license to display the preprint in perpetuity. It is made available under aCC-BY 4.0 International license.

with JF635i-ligand (cyan) for 24 hours. Nuclei visualized with DAPI. Scale bar: 10 µm. **(c)** Quantification of JF635i ligand fluorescence intensity in extracellular HaloTag (blue) and cytosolic HaloTag (orange) cells. Data are mean ± SEM, extracellular n = 124, cytosolic n = 153 cells, ****$p < 0.0001$, Welch's unpaired t test. **(d)** Relationship between HaloTag receptor expression (mScarlet fluorescence) and JF635i ligand fluorescence intensity in extracellular HaloTag and cytosolic HaloTag conditions. Data points represent single cells transfected with extracellular HaloTag (blue, n=124) or cytosolic HaloTag (orange, n=153). Linear regression for extracellular HaloTag: y=0.3449x+0.0367, r^2=0.30. Linear regression for cytosolic HaloTag: y=0.0013x+ 0.0002, r^2=0.66. **(e)** 63x magnification confocal images of HEK cells transfected with extracellular HaloTagTM-GFP (magenta) incubated with JF549i-ligand (cyan) for 0.5 or 24 hours. Nuclei visualized with DAPI. Scale bar: 10 µm. **(f)** Relationship between HaloTag receptor expression (mScarlet fluorescence) and JF635i ligand fluorescence intensity in extracellular HaloTag and cytosolic HaloTag conditions. Data points represent single cells incubated with ligand for 0.5h (black, n=186) or 24h (teal, n=91). Linear regression for 0.5h incubation condition: y=0.9323x+0.0431, r^2=0.74. Linear regression for 24h incubation condition: y=0.9388x+ 0.1520, r^2=0.50. **(g)** Slidescanner image of periventricular brain region of a C57Bl6 mouse injected with AAV-HaloTagTM-mScarlet. Nuclei, vessels, and HaloTagTM-mScarlet all shown in grayscale. Yellow arrow points to the lateral ventricle choroid plexus, which is not transduced.

bioRxiv preprint doi: https://doi.org/10.1101/2025.10.30.685531; this version posted October 31, 2025. The copyright holder for this preprint (which was not certified by peer review) is the author/funder, who has granted bioRxiv a license to display the preprint in perpetuity. It is made available under aCC-BY 4.0 International license.

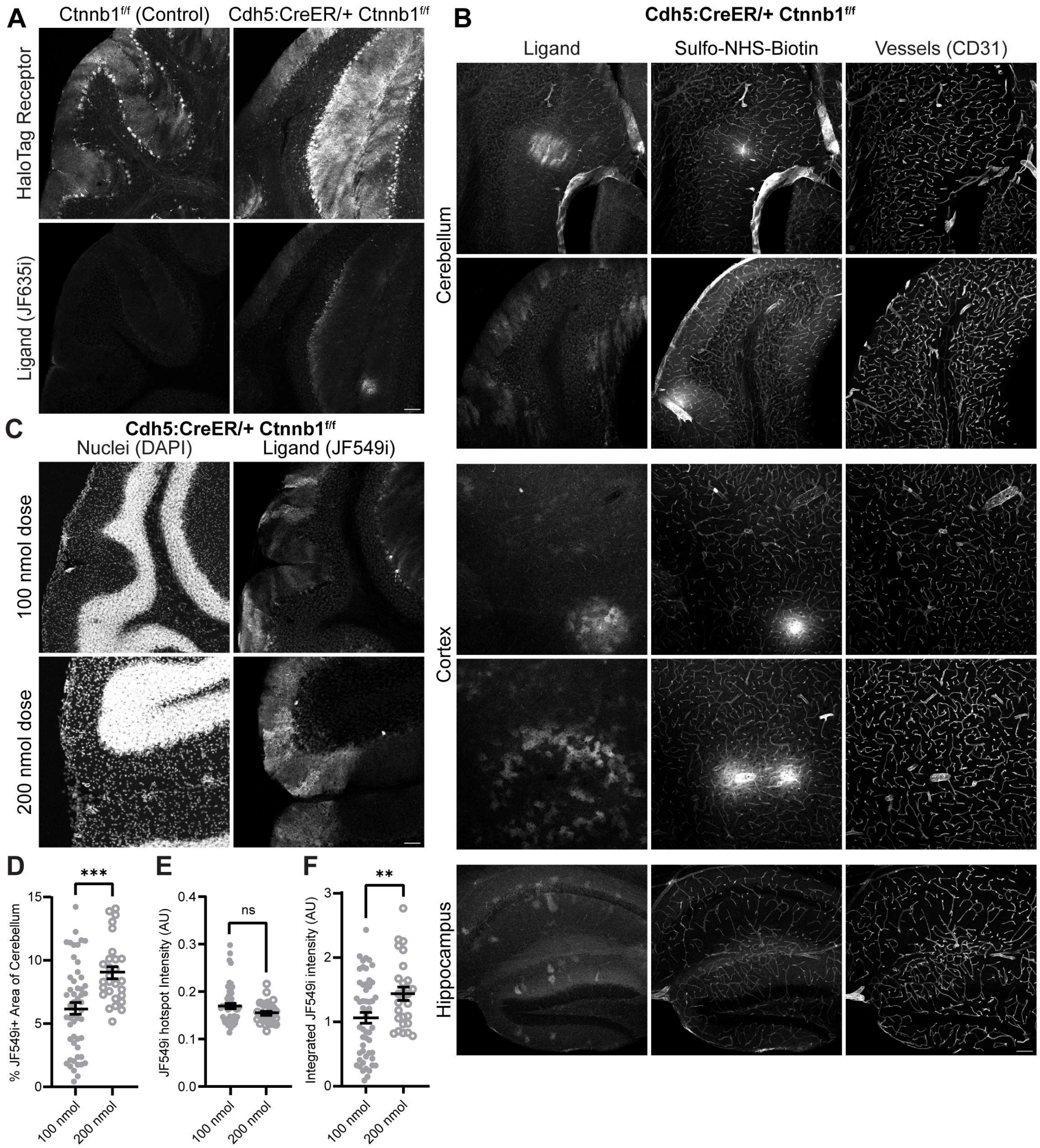

Supplementary Figure 2. Further characterization of HaloTrace in Ctnnb1 knockdown.

(a) Representative 10x magnification confocal images of HaloTrace permeability assay in Cdh5:CreER/+; Ctnnb1$^{f/f}$ and control Ctnnb1$^{f/f}$ cerebellums processed by dropfixation without transcardial perfusion. **(b)** Representative 10x magnification confocal images of Cdh5:CreER/+; Ctnnb1$^{f/f}$ brains with BBB leakage assayed by HaloTrace and sulfo-NHS-biotin. Vessels

bioRxiv preprint doi: https://doi.org/10.1101/2025.10.30.685531; this version posted October 31, 2025. The copyright holder for this preprint (which was not certified by peer review) is the author/funder, who has granted bioRxiv a license to display the preprint in perpetuity. It is made available under aCC-BY 4.0 International license.

visualized with anti-CD31 antibody. **(c)** Representative 10x magnification confocal images of ligand deposition in Cdh5:CreER/+; Ctnnb1$^{f/f}$ cerebellum after injection of 100 nmol or 200 nmol JF549i ligand. Scale bar: 100µm. **(d)** Quantification of total JF549i-ligand containing area of cerebellum. *** indicates p=0.002, unpaired t test. **(e)** Quantification of JF549i+ area mean intensity. ns, nonsignificant, p=0.055, unpaired t test. **(f)** Quantification of integrated intensity of JF549+ positive area. ** indicates p=0.008, unpaired t test. For b-d, data points represent individual images from n=3 mice per dosage group. Filled gray circles: 100 nmol dose. Empty gray circles: 200 nmol dose. Data for the 100 nmol condition were also used in the analysis of Figure 4. For a,c,d, Scale bar: 100 µm.

bioRxiv preprint doi: https://doi.org/10.1101/2025.10.30.685531; this version posted October 31, 2025. The copyright holder for this preprint (which was not certified by peer review) is the author/funder, who has granted bioRxiv a license to display the preprint in perpetuity. It is made available under aCC-BY 4.0 International license.

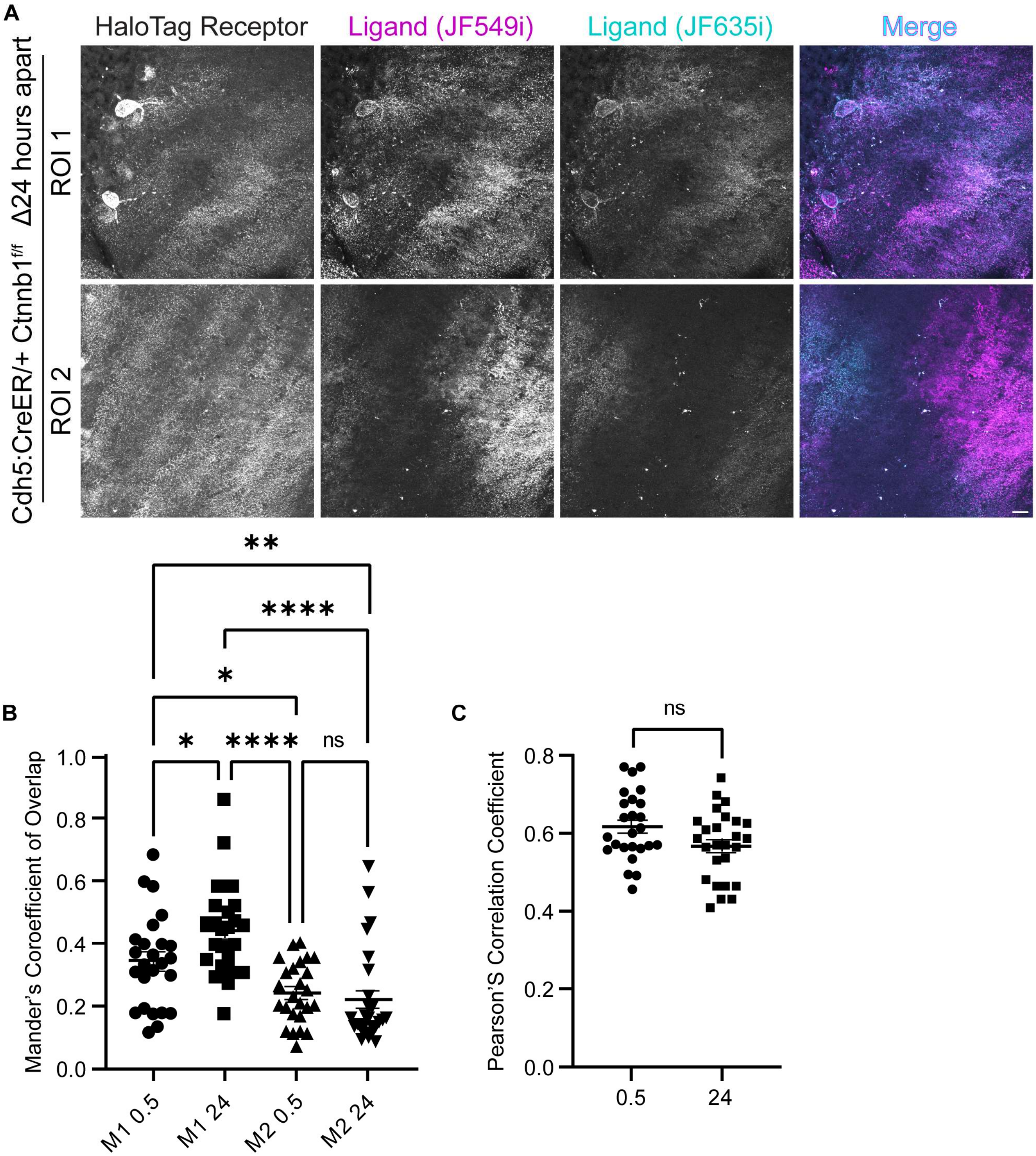

Supplementary Figure 3. Intensity-based colocalization analyses of the pattern of JF635i- and JF549i-ligand.

(a) 40x magnification confocal images of HaloTag receptor, JF549i-ligand (magenta in merge) and JF635i-ligand (cyan in merge) in Cdh5:CreER/+; Ctnnb1$^{f/f}$ cerebellum. The top row shows

bioRxiv preprint doi: https://doi.org/10.1101/2025.10.30.685531; this version posted October 31, 2025. The copyright holder for this preprint (which was not certified by peer review) is the author/funder, who has granted bioRxiv a license to display the preprint in perpetuity. It is made available under aCC-BY 4.0 International license.

an example of highly colocalized ligands whereas the bottom row shows an example of relatively distinct ligand hotspots. Scale bar: 20 µm. **(b)** Manders colocalization analysis. M1 corresponds to the fraction of JF549i overlapping JF635i in the Δ0.5h or Δ24h conditions; M2 corresponds to the fraction of JF635i overlapping JF549i. *$p<0.05$, ****$p<0.0001$, ns: nonsignificant, ANOVA with Tukey's correction for multiple comparisons. **(c)** Pearson's correlation coefficient as a measure of JF635i and JF549i-ligand overlap in the Δ0.5h or Δ24h conditions. ns: nonsignificant, student's t test. $p=0.055$. For both analyses, $n = 25$ images in Δ0.5h group and $n = 26$ for the Δ24h group. Note that Pearson's correlation coefficient can overestimate intensity correlation in samples with high background, like these, because it does not exclude background pixels from the analysis.
